# Dr. Brown's Opening Address

**Published:** 1844-09

**Authors:** 


*Dr. Brown's Opening Address.*—We delayed the publication of the present number of the Journal, nearly three weeks, in order to obtain the address delivered by Dr. S. Brown, before the American Society of Dental Surgeons, at its last annual meeting, and not receiving it, after having waited so long, we were reluctantly compelled to omit its insertion. We presume the reason of our not having yet received it, is owing to the fact, it having been delivered extempore, that the doctor has not yet had leisure to write it out. We hope, however, to be able to give it in the next number. The address was highly interesting, and listened to with great attention by all of the members present on the occasion of its delivery.—*Balt. Ed.*

